# When a Seizure Reveals More Than a Hemorrhage: When to Suspect Cerebral Cavernous Malformations

**DOI:** 10.7759/cureus.117023

**Published:** 2026-09-27

**Authors:** Fernando Uribe-Hernández, Jesus Reyes-Reyes

**Affiliations:** 1 Department of Medicine, National Autonomous University of Mexico, Iztacala Faculty of Higher Studies, Tlalnepantla, MEX; 2 Department of Neurosurgery, Instituto de Seguridad Social del Estado de México y Municipios (ISSEMYM), Regional Hospital Tlalnepantla, Tlalnepantla, MEX

**Keywords:** cavernomas, cerebral cavernoma, cerebral cavernous malformation, frontal cavernoma, magnetic resonance imaging

## Abstract

Cerebral cavernous malformations (CCMs) are low-flow vascular malformations that may be asymptomatic or present with seizures, hemorrhage, headache, or focal neurological deficits. Most are sporadic and solitary, whereas familial CCMs are associated with multiple lesions and autosomal-dominant inheritance. Distinguishing between these forms is clinically relevant for long-term management. We present a 33-year-old woman who presented with generalized tonic-clonic seizures following sleep deprivation. Computed tomography (CT) initially suggested a left frontal intraparenchymal hemorrhage. Magnetic resonance imaging (MRI) subsequently revealed two left frontal Zabramski type II CCMs. Electroencephalography showed cortical dysfunction with paroxysmal activity and contralateral propagation. Antiseizure treatment with phenytoin was initiated and later supplemented with levetiracetam. The patient remained asymptomatic and was referred for tertiary evaluation. This case highlights the diagnostic limitations of CT and the importance of MRI for CCM characterization and detection of multiple lesions. The presence of two cavernomas and a potentially relevant family history raises suspicion for familial CCM, although these findings alone do not establish a hereditary diagnosis. Genetic evaluation and detailed family assessment should therefore be considered. Treatment should be individualized; conservative management is appropriate in selected stable patients, whereas microsurgery and stereotactic radiosurgery are reserved for specific clinical scenarios.

## Introduction

Cerebral cavernous malformations (CCMs), also known as cavernomas or cavernous angiomas, are benign, low-flow vascular malformations composed of dilated vascular spaces lined by endothelial cells and that don’t interfere with the neural parenchyma [1-4]. They are considered angiographically occult and are most accurately characterized by magnetic resonance imaging (MRI), particularly with susceptibility-sensitive sequences such as T2*-weighted gradient-recalled echo (GRE) and susceptibility-weighted imaging (SWI) [2-3,5-6]. CCMs occur in approximately 0.5% of the general population and can affect patients at any age, although they are commonly recognized during adulthood [1-4].

Their clinical spectrum includes asymptomatic incidental lesions, seizures, headaches, focal neurological deficits, and symptomatic intracranial hemorrhage [1-3,7]. Most CCMs are sporadic and solitary, whereas familial disease is characterized by autosomal-dominant inheritance with incomplete penetrance and a tendency toward multiple lesions, principally associated with pathogenic variants in CCM1/KRIT1, CCM2, and CCM3/PDCD10 [2-4,8]. 

We present a 33-year-old woman who developed generalized tonic-clonic seizures and whose initial CT suggested a left frontal intraparenchymal hemorrhage, while subsequent MRI demonstrated two left frontal Zabramski type II CCMs. The multiplicity of lesions and the patient's family history make the distinction between sporadic and familial disease particularly relevant to her long-term evaluation and management.

## Case presentation

This case deals with a 33-year-old female patient. The current illness began after a period of sleep deprivation, when the patient experienced a first episode of generalized tonic-clonic seizure witnessed by relatives. The event lasted approximately three minutes and was accompanied by urinary incontinence and tongue biting. A postictal period of approximately 30 minutes followed, characterized by somnolence, headache, asthenia, adynamia, and up to three episodes of vomiting. She was subsequently transferred to our emergency department for evaluation and treatment.

Her personal history was notable for traumatic brain injuries at 7 and 17 years of age, multiple migraine episodes since adolescence, and one cesarean section. She denied substance abuse, chronic degenerative diseases, and allergies. Family history included a maternal grandfather with unspecified epilepsy and a mother with unspecified cerebrovascular disease. The family history was explored further, but the patient could not recall additional relevant diagnoses or clinical details among her relatives.

Regarding the timeline and initial diagnostic evaluation, a head computed tomography (CT) scan was performed, revealing a hyperdense area (average attenuation of 64 HU) of 16 x 15 x 14 mm in the left middle frontal gyrus, a grade II sellar arachnoidocele, and a right septal deviation (Figure 1). This initially posed the diagnostic challenge of differentiating between a probable left frontal intraparenchymal hemorrhage and a space-occupying lesion. After three days, she was discharged from the emergency department as she did not experience new seizures, and an initial antiseizure treatment with phenytoin was prescribed, along with requests for an electroencephalogram (EEG) and brain magnetic resonance imaging (MRI).

**Figure 1:**
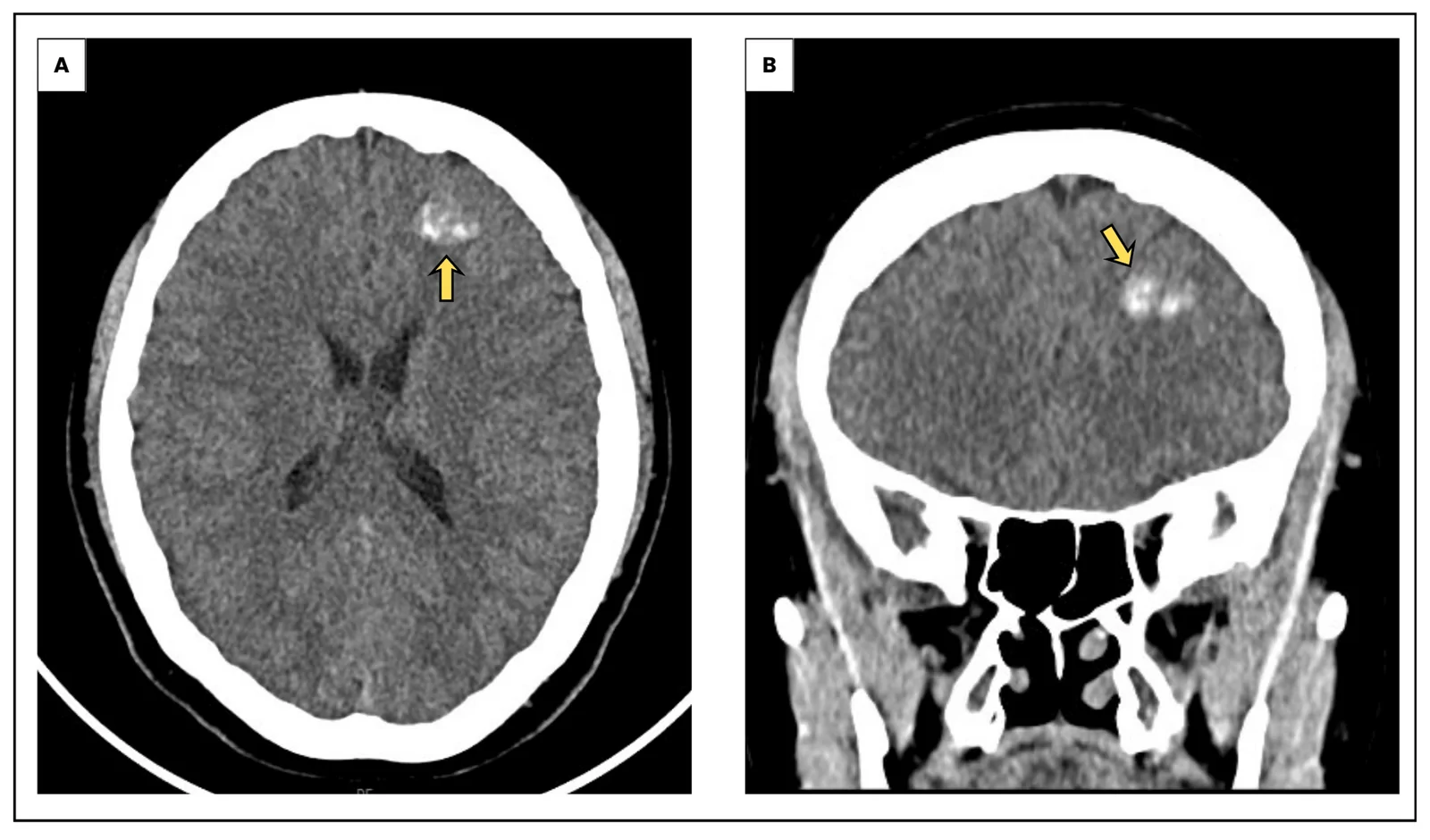
(A) Axial head CT, (B) coronal head CT Both showing a hyperdense intraparenchymal lesion in the left middle frontal gyrus (yellow arrows), measuring approximately 16 x 15 x 14 mm, with a mean attenuation of 64 HU.

Following her discharge, she was evaluated for the first time in the neurosurgery outpatient consultation. On physical and neurological examination, the patient had a Glasgow Coma Scale score of 15, with intact higher mental functions, cranial nerves, motor skills, and sensitivity. A notable finding during the tandem gait assessment was a slight loss of balance control, and the patient reported occasional feelings of instability; however, a clear cerebellar syndrome was not identified.

Subsequently, the diagnostic approach was complemented with an EEG and brain MRI with MR angiography. The EEG showed slowing of the background activity (theta waves) and paroxysms of slow spike-waves in the right frontocentro-temporal region that propagated to the contralateral hemisphere, integrating the diagnosis of bilateral cortical irritation and cerebral functional alteration (Figure 2).

**Figure 2:**
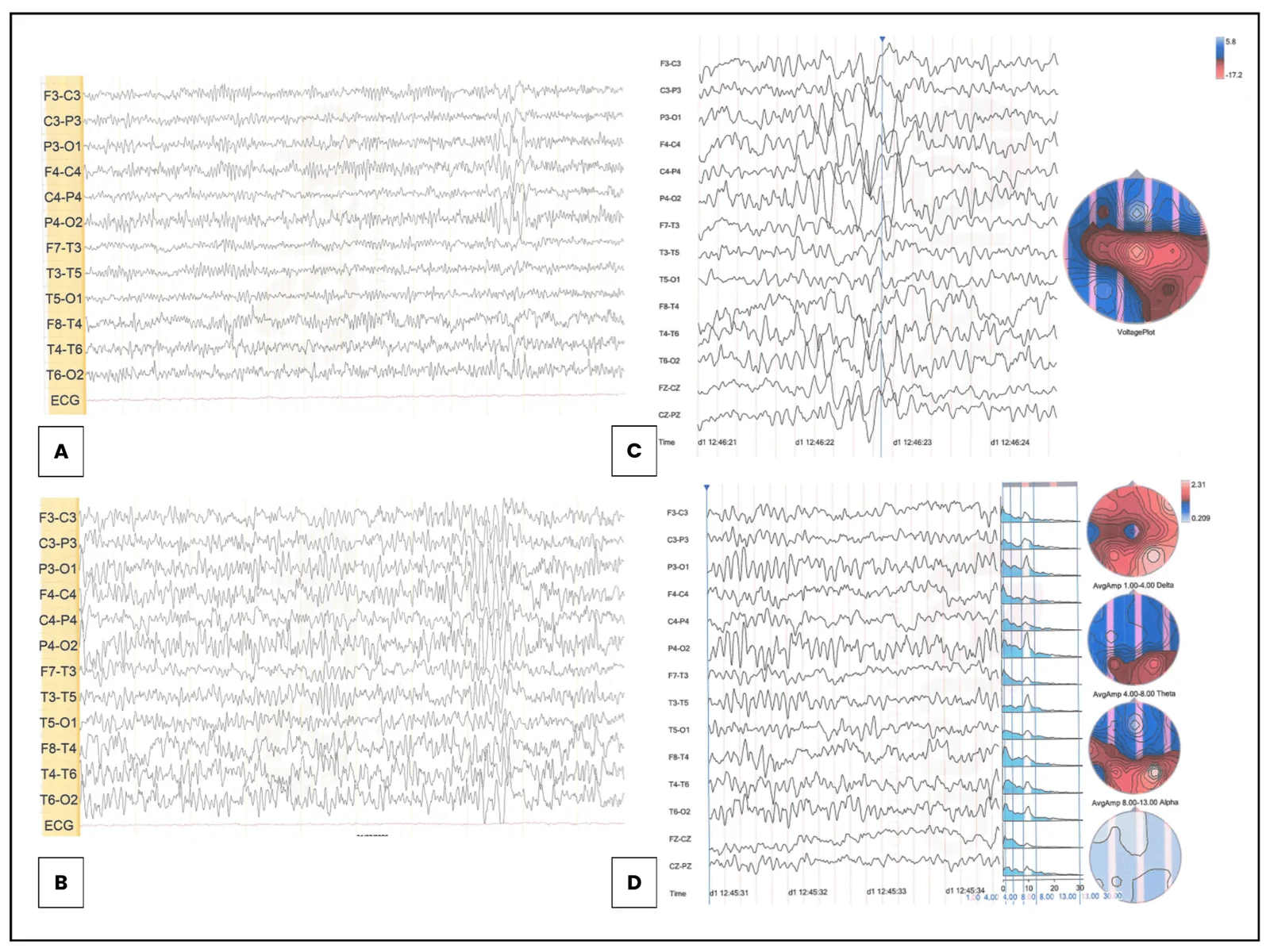
Electroencephalography and quantitative brain mapping (A) and (B) Digital resting-state electroencephalography showing poorly organized alpha rhythm and diffuse slowing of the background activity with theta-range activity (4-7 Hz). Focal paroxysms consisting of sharp waves and slow angular spike-and-wave complexes are observed in the right frontocentrotemporal region, with ipsilateral and contralateral propagation, consistent with moderate focal cortical irritation. (C) and (D) Quantitative brain mapping demonstrating globally increased absolute and relative theta power, with decreased relative alpha power and reduced mean frequency, findings consistent with functional cerebral alteration due to slowing and disorganization of background activity.

The MRI accurately defined the etiology by revealing two left frontal lesions: one measuring 16.5 x 20 x 18 mm in the superior frontal gyrus and another measuring 7.2 x 11.5 x 10 mm in the medial rectus gyrus (Figure 3). A complete MRI protocol, including susceptibility-weighted imaging (SWI), was requested; however, the SWI sequence was not available at our center at the time of acquisition. The available sequences included T2* gradient-recalled echo (GRE), T2 fluid-attenuated inversion recovery (FLAIR), T2 fast spin echo (FSE), diffusion-weighted imaging (DWI), and apparent diffusion coefficient (ADC). Both lesions were compatible with cavernous angiomas (cavernomas) type II according to the Zabramski classification. The MRI findings therefore favored CCMs over the intraparenchymal hemorrhage initially suggested by CT. 

**Figure 3:**
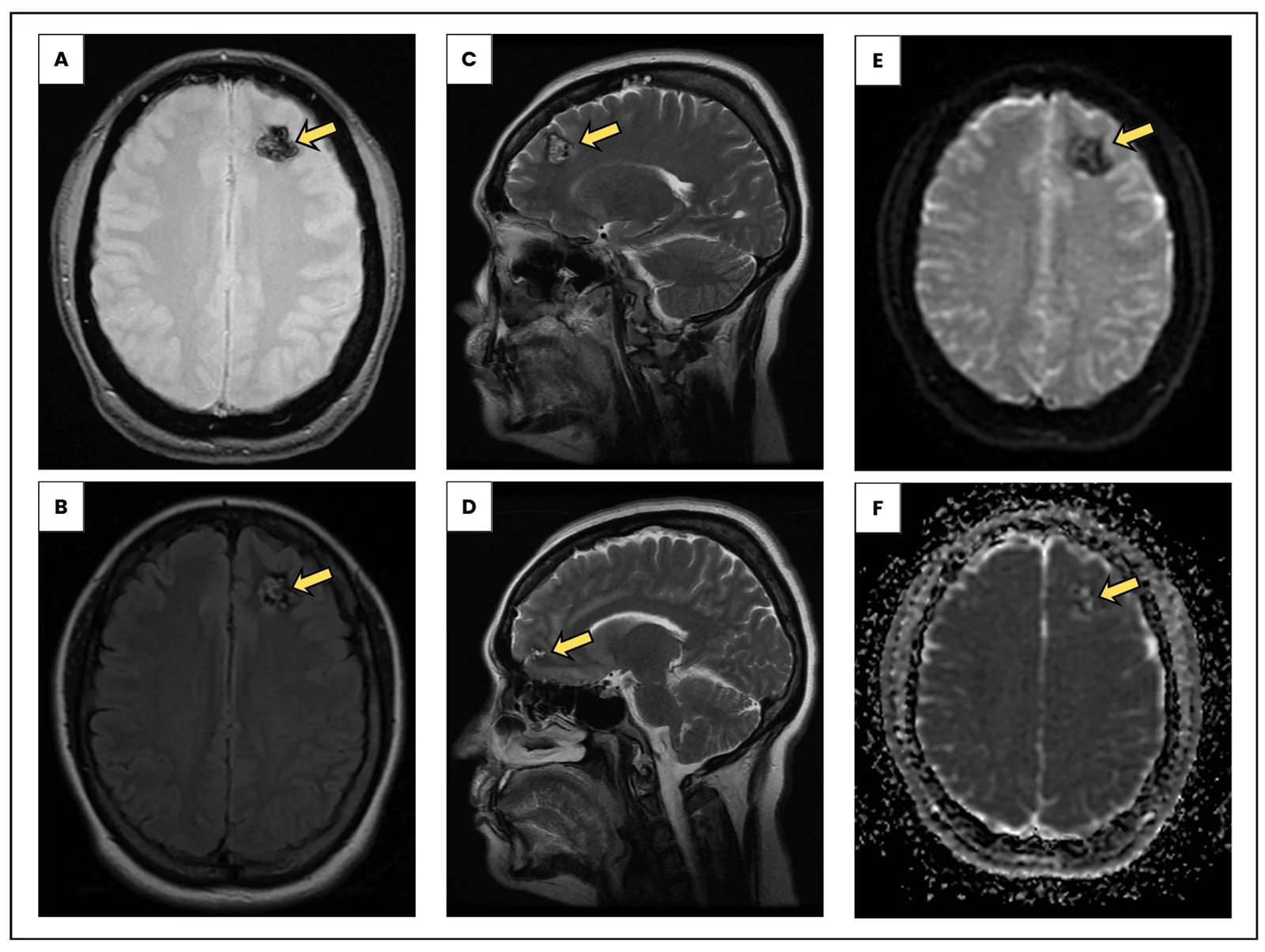
Brain MRI (A) Axial T2* GRE sequence showing the larger cavernous malformation in the left superior frontal gyrus (yellow arrow), characterized by heterogeneous signal intensity and marked magnetic susceptibility (“blooming”). (B) Axial T2 FLAIR sequence demonstrating the same lesion (yellow arrow). (C) Sagittal T2 FSE sequence showing the larger lesion at the most rostral aspect of the left superior frontal gyrus (yellow arrow). D) Sagittal T2 FSE sequence demonstrating a second, smaller lesion in the rostral and medial aspect of the left straight gyrus (yellow arrow). (E) DWI and (F) ADC map of the larger lesion (yellow arrows), with no evidence of restricted diffusion. Both lesions exhibit imaging characteristics consistent with Zabramski type II cavernous malformations, without surrounding vasogenic edema. Although SWI was requested as part of the MRI protocol, it was not available at the time of acquisition; therefore, T2* GRE was the available susceptibility-sensitive sequence. Abbreviations: GRE, gradient-recalled echo; T2 FLAIR, T2-weighted fluid-attenuated inversion recovery; T2 FSE, T2-weighted fast spin echo; DWI, diffusion-weighted imaging; ADC, apparent diffusion coefficient.

As a therapeutic intervention, because the EEG demonstrated persistent cortical irritation despite the use of phenytoin, a modification in management was justified by adding levetiracetam as a concomitant antiseizure therapy. The management was supplemented with preventive measures and follow-up. At the three-month follow-up, the patient remained asymptomatic, reported good adherence to antiseizure therapy, and had no recurrent seizures. No genetic testing or evaluation of family members was performed at our center during this period. Our institution is a regional second-level care center in the State of Mexico serving state workers; molecular genetic testing and specialized tertiary neurogenetic evaluation were not available locally. Therefore, the patient was referred to a tertiary-care center for further evaluation and definitive management, with a recommendation for molecular genetic testing/mapping to clarify whether the disease represented familial cerebral cavernous malformation (FCCM) or a sporadic form, as well as family assessment and counseling.

## Discussion

Definition, epidemiology, and pathophysiology

CCMs are non-neoplastic, low-flow vascular malformations characterized by closely packed dilated sinusoidal vascular spaces lined by endothelial cells, with little or no intervening neural parenchyma [1-5]. Their walls lack the normal smooth-muscle and elastic components of cerebral vessels, contributing to vascular fragility and susceptibility to repeated intralesional bleeding and thrombosis [2,3,5]. These recurrent hemorrhagic and thrombotic events lead to progressive deposition of hemosiderin, calcification, and gliotic changes in and around the lesion [1,5,6].

CCMs are detected in approximately 0.5% of the general population, with published prevalence estimates ranging broadly depending on the population and imaging method used [1-4]. They may occur anywhere in the central nervous system, although supratentorial lesions predominate [1-3,6]. Clinical manifestations are heterogeneous, and patients may remain asymptomatic or present with seizures, headaches, focal neurological deficits, or hemorrhage [1-3,7].

The pathophysiological basis of CCM-related epilepsy is particularly relevant to the present case. Repeated microhemorrhage leads to perilesional hemosiderin deposition and gliosis, while blood-breakdown products may promote oxidative stress and neuronal hyperexcitability [1,7,9]. In a cohort of 199 patients with pathologically confirmed CCMs, Zhang et al. found that CCM-related epilepsy was associated particularly with cortical involvement and a surrounding hemosiderin rim, while focal-to-bilateral tonic-clonic seizures were the most common seizure type [7]. These findings are relevant to this patient, who presented with a generalized tonic-clonic seizure and had frontal cortical lesions.

Sporadic and familial cerebral cavernous malformations

CCMs may occur as sporadic, familial, or radiation-associated disease [2-4,8]. Sporadic lesions most commonly present as a single lesion, whereas familial disease typically demonstrates multiple lesions and may be accompanied by the progressive appearance of additional CCMs over time [2,3,8]. Familial disease follows an autosomal-dominant inheritance pattern with incomplete and age-dependent penetrance and is principally caused by pathogenic variants affecting CCM1/KRIT1, CCM2, or CCM3/PDCD10 [3,4,8] (Table 1).

**Table 1:** Comparison of sporadic and familial cerebral cavernous malformations Typical phenotype and inheritance [2-4,6]; main genetic alterations [2,4-6]; multiplicity [2-4,6]; development of new lesions [2-4,6]; family history and criteria for suspecting familial disease [2,4,6]; genetic counseling/testing [4,6]. Abbreviations: CCM, cerebral cavernous malformation; FCCM, familial cerebral cavernous malformation.

Feature	Sporadic CCM	Familial CCM
Typical phenotype	Usually presents as a solitary lesion	Characteristically associated with multiple lesions
Inheritance	Generally not associated with a germline hereditary pattern	Autosomal-dominant inheritance with incomplete penetrance
Main genetic alterations	Somatic alterations in CCM-related pathways may occur	Germline pathogenic variants, principally involving CCM1/KRIT1, CCM2, and CCM3/PDCD10
Multiplicity	Usually single; multiple lesions may occasionally occur	Multiple lesions are a characteristic imaging feature
Development of new lesions	May occur in selected cases	New lesions may develop over time, reflecting the dynamic nature of familial disease
Family history	Usually absent	A positive family history supports the diagnosis, although incomplete penetrance may result in clinically unrecognized affected relatives
When to suspect familial disease	Multiplicity is not sufficient by itself to establish familial disease	Multiple CCMs, particularly together with a suggestive family history, should raise suspicion for FCCM
Genetic counseling/testing	Usually not necessary for an isolated typical sporadic lesion	Should be considered when multiple lesions or a suggestive pedigree are present

The molecular distinction between the two forms is increasingly understood. Familial disease commonly begins with a germline loss-of-function variant followed by a somatic second hit affecting the remaining normal allele in susceptible endothelial cells. This mechanism contributes to clonal expansion of CCM-deficient endothelial populations and subsequent lesion formation [2,8]. Sporadic lesions may also involve somatic alterations in CCM-associated pathways, illustrating that familial and sporadic disease can converge on common endothelial signaling mechanisms despite having different genetic origins [2,4,5,8].

Multiplicity is therefore an important clinical clue but not a definitive diagnostic criterion for familial disease. Sporadic patients can occasionally present with more than one lesion, including lesions associated with developmental venous anomalies [3-6]. Consequently, multiple lesions should trigger consideration of a hereditary syndrome rather than being automatically classified as FCCM.

Clinical presentation and CCM-related epilepsy

The most common clinical manifestation of supratentorial CCMs is seizure activity [1-3,7,9]. Zhang et al. reported that focal-to-bilateral tonic-clonic seizures were the most frequent seizure type among patients with CCM-related epilepsy, while cortical involvement and a hemosiderin rim were important risk factors [7].

Our patient's generalized tonic-clonic seizure after sleep deprivation is compatible with the clinical spectrum of supratentorial CCMs. Nevertheless, the relationship between her EEG findings and the structural lesions deserves particular attention. The MRI demonstrated two left frontal lesions, whereas EEG abnormalities were predominantly recorded in the right frontocentrotemporal region with contralateral propagation.

This discordance does not exclude CCM-related epilepsy, but it prevents a straightforward assumption that either left frontal lesion is definitively the seizure-onset zone. Establishing causality becomes particularly important when surgery is contemplated because surgical decision-making for CCM-related epilepsy should ideally be based on sufficient electroclinical and anatomical concordance [5,7,10].

Dulamea and Ene emphasize that after a first seizure considered related to a CCM, antiseizure medication is appropriate, whereas prophylactic antiseizure therapy is not indicated for incidentally discovered CCMs without prior seizures [5]. Thus, the initial pharmacological strategy in this case is consistent with the management framework described in the literature.

Neuroimaging

Magnetic resonance imaging (MRI) is the preferred imaging modality for cerebral cavernous malformations (CCMs), with greater sensitivity than computed tomography (CT), particularly for small or chronic lesions. Susceptibility-sensitive sequences, including gradient-echo and susceptibility-weighted imaging, are especially useful for detecting hemosiderin and additional lesions [1,2,6]. In the present case, SWI was requested as part of the MRI protocol but was not available at our center at the time of acquisition. The available T2* GRE sequence provided susceptibility information; however, the absence of SWI may reduce sensitivity for small additional lesions or subtle hemosiderin deposition.

Based on MRI characteristics and pathological correlation, Zabramski et al. classified CCMs into four types [6,11] (Table 2). Type I represents subacute hemorrhage, with a hyperintense T1-weighted core related to methemoglobin and variable T2 signal depending on hemorrhage evolution. Type II shows the classic heterogeneous or “popcorn” appearance, with mixed signal intensity on T1 and T2 and a hypointense hemosiderin rim, reflecting hemorrhage and thrombosis of different ages. Type III corresponds to chronic hemorrhage and is predominantly iso- or hypointense on T1 and markedly hypointense on T2 and gradient-echo sequences because of hemosiderin deposition. Type IV lesions are poorly visualized on conventional T1- and T2-weighted images and appear as small punctate hypointense foci on gradient-echo sequences [6,11]. 

**Table 2:** Zabramski MRI classification of cerebral cavernous malformations Type I characteristics [11]; Type II characteristics [6,11]; Type III characteristics [6,11]; Type IV characteristics [6,11]. Abbreviations: CCM, cerebral cavernous malformation

Type	T1-weighted MRI	T2-weighted MRI	Gradient-echo / susceptibility sequences	Pathological correlate	Typical interpretation
Type I	Hyperintense core	Initially hyperintense; may become heterogeneous as blood products evolve	May demonstrate susceptibility from blood products	Subacute hemorrhage with methemoglobin and subsequent blood-product degradation	Recent/subacute hemorrhagic lesion
Type II	Mixed/reticulated signal	Mixed/reticulated core with hypointense hemosiderin rim	Marked susceptibility effect	Loculated hemorrhage and thrombosis of varying ages, surrounded by gliosis and hemosiderin; calcification may occur in larger lesions	Classic CCM appearance; mixed-age hemorrhage
Type III	Isointense to hypointense	Markedly hypointense with hypointense rim	Pronounced susceptibility effect	Chronic resolved hemorrhage with hemosiderin deposition within and around the lesion	Chronic hemorrhagic lesion
Type IV	Poorly visualized or occult	Poorly visualized or occult	Punctate hypointense foci	Minute hemosiderin deposits; some lesions in the original series were pathologically confirmed as capillary telangiectasias	Very small/occult lesion; best detected by susceptibility imaging

Type II is the most characteristic pattern and was the classification assigned to both lesions in our patient. In the original familial CCM cohort, Type I and II lesions were more frequently associated with symptoms and recent hemorrhagic activity, whereas Type III and IV lesions were more often clinically silent [11]. 

In the present case, the diagnosis was a challenge. The initial CT demonstrated a hyperdense left frontal lesion with a mean attenuation of approximately 64 HU, making intraparenchymal hemorrhage a reasonable initial consideration. MRI subsequently demonstrated two left frontal Type II CCMs; the revelation of lesion multiplicity is relevant when considering possible familial disease. 

Natural history and hemorrhagic risk

The natural history of CCMs is variable. Lesions may remain stable, change in volume and MRI signal, thrombose, regress, enlarge, or undergo symptomatic hemorrhage [1,3-6].

The risk of symptomatic hemorrhage is strongly influenced by the clinical context and lesion location. Patients presenting with a previous symptomatic hemorrhage have a higher subsequent risk than patients with incidentally discovered, previously unbled lesions [2,4,5]. Brainstem lesions also carry greater clinical consequences because relatively small hemorrhages may cause substantial neurological deficits [2,3,5].

The present patient does not have a documented symptomatic CCM hemorrhage. Although the initial CT raised concern for intraparenchymal hemorrhage, the subsequent MRI established the lesions as Zabramski Type II CCMs, and there was no clinical syndrome clearly attributable to an acute hemorrhage. This distinction is important because a hyperdense lesion on CT should not automatically be classified as a symptomatic CCM hemorrhage without appropriate clinical and MRI correlation [1,6].

Treatment

Treatment of CCMs is individualized and generally falls into three broad categories: conservative management, microsurgical resection, and stereotactic radiosurgery [2-5,10]. The decision depends on symptoms, lesion location, prior hemorrhage, eloquence, accessibility, seizure control, lesion multiplicity, comorbidities, and the anticipated morbidity of intervention [4,5] (Table 3).

**Table 3:** Therapeutic approach according to clinical scenario Incidental/asymptomatic CCM [2-5]; first CCM-related seizure and seizure controlled with medication [5,7,9]; medically refractory CCM-related epilepsy [2,5,7,10]; symptomatic surgically accessible CCM [2-5]; deep/eloquent lesion with high surgical risk [2-5]; surgically inaccessible symptomatic CCM [2-3,5,10]; multiple asymptomatic CCMs [2-4,6]; multiple CCMs with suggestive family history [2,4,6,11].

Clinical scenario	Preferred approach	Rationale / Supporting references
Incidental or asymptomatic CCM	Conservative management	Prophylactic surgery is generally not recommended when the expected treatment morbidity exceeds the natural-history risk
First CCM-related seizure	Antiseizure medication	Medical treatment is generally the initial approach after a first seizure attributed to a CCM
Seizure controlled with medication	Continue conservative management	Surgery is not automatically indicated when seizures are adequately controlled
Medically refractory CCM-related epilepsy	Microsurgical evaluation/resection	Surgery provides lesion-directed treatment and substantial seizure-control rates in appropriately selected patients
Symptomatic, surgically accessible CCM	Microsurgical resection may be considered	Benefit may outweigh operative morbidity when symptoms are attributable to an accessible lesion
Deep or eloquent lesion with high surgical risk	Individualized management	Observation may be preferable when surgical morbidity is substantial
Surgically inaccessible symptomatic CCM	Selected stereotactic radiosurgery	SRS may be considered when microsurgical treatment carries excessive risk
Multiple asymptomatic CCMs	Conservative management	Multiplicity alone is not an indication for prophylactic resection
Multiple CCMs + suggestive family history	Conservative/lesion-specific treatment + genetic evaluation	In the present case, the right frontocentrotemporal EEG abnormalities were not concordant with the two left frontal CCMs identified on MRI, so a definitive epileptogenic focus could not be established. Immediate surgical decision-making was therefore deferred pending tertiary evaluation

Conservative management

Conservative management is generally preferred for asymptomatic or incidentally discovered CCMs, particularly when the lesion is located in an area where intervention carries substantial neurological risk. Management consists primarily of clinical follow-up, treatment of symptoms, antiseizure medication when seizures occur, and individualized radiological surveillance [2-5]. 

The current treatment literature emphasizes that follow-up should be individualized rather than rigidly standardized, with particular attention to patients with multiple lesions, familial CCM, and lesions located in high-risk anatomical regions [5].

For CCM-related epilepsy, antiseizure medication represents the initial therapeutic approach after a first seizure [5,7]. This is especially relevant to the present patient, who remained asymptomatic after pharmacological treatment and had no documented recurrent clinical seizure during the described follow-up.

Microsurgical resection

Surgical treatment becomes more compelling when a CCM is symptomatic and surgically accessible, particularly when symptoms are attributable to the lesion and the anticipated operative morbidity is acceptable [2,3,5]. In CCM-related epilepsy, microsurgical resection is especially relevant when seizures persist despite adequate antiseizure therapy or when the epileptogenic relationship between the lesion and seizure onset is well established [5,7,10].

Gao et al.'s systematic review and meta-analysis of 45 studies found an overall seizure-control rate of 79% after neurosurgery, compared with 49% after radiosurgery, although neurosurgery was associated with a higher rate of permanent morbidity [10]. These findings favor microsurgery for appropriately selected epileptogenic CCMs but also emphasize that patient selection is critical.

Pereira et al. further demonstrated the importance of anatomical planning in surgery for cavernous malformations, describing the superior frontal sulcus as a potential non-eloquent corridor for selected lesions of the internal capsule [12]. While their specific anatomical scenario differs from the present case, the study reinforces the principle that surgical accessibility and the relationship between the lesion and eloquent structures must be evaluated individually.

Importantly, surgery is not routinely recommended for asymptomatic CCMs merely because they are multiple [2,3,5]. In the current patient, the absence of recurrent clinical seizures, absence of a documented symptomatic hemorrhage, preserved neurological status in the three-month follow-up, and lack of a clearly concordant epileptogenic focus make immediate microsurgical resection less compelling.

Stereotactic radiosurgery

Stereotactic radiosurgery (SRS) may be considered in selected symptomatic CCMs when the lesion is surgically inaccessible or when microsurgical morbidity is considered unacceptably high [2,3,5]. Ganz describes both microsurgery and radiosurgery as treatment options, particularly in the context of lesion location and the distinct behavior of brainstem lesions [3].

However, SRS remains controversial. Dulamea and Ene emphasize the limited long-term evidence and the potential for treatment-related hemorrhage, edema, cyst formation, and delayed radiation toxicity [5]. In addition, the potential development of radiation-induced CCMs is particularly relevant in patients with familial disease, in whom multiple lesions may already reflect an underlying biological predisposition [5,8].

The systematic review by Gao et al. found lower seizure-control rates after radiosurgery than after neurosurgery, although the included evidence was observational and heterogeneous [10]. Therefore, SRS should not be regarded as an equivalent substitute for microsurgical resection in an accessible, symptomatic CCM.

Familial evaluation as a component of management

In this case, determining whether the disease is sporadic or familial is not merely a genetic curiosity. It may influence surveillance, counseling, interpretation of future MRI findings, and assessment of relatives [2,4,8]. Because molecular genetic testing was not available at our center, no genetic study was performed during the reported three-month follow-up. The patient was referred to tertiary care for genetic evaluation and family assessment.

The presence of two lesions should therefore prompt careful pedigree reconstruction and consideration of genetic counseling. A pathogenic germline variant in KRIT1/CCM1, CCM2, or PDCD10/CCM3 would support FCCM and have implications for family assessment [2,4,8]. In the present case, no family members were evaluated locally because the patient was referred to tertiary care; further family assessment and counseling were recommended after referral.

Conversely, an absence of a documented family history cannot by itself exclude FCCM because incomplete penetrance means that affected relatives may remain asymptomatic [8]. In the present patient, the reported epilepsy in her maternal grandfather and unspecified cerebrovascular disease in her mother therefore deserve clarification but should not be retrospectively reclassified as CCM manifestations without evidence. 

This distinction strengthens the scientific value of the case: the diagnosis of multiple CCMs is established, whereas the sporadic-versus-familial nature of the disease remains an important unresolved clinical question.

## Conclusions

This case illustrates how a frontal CCM may initially mimic intraparenchymal hemorrhage on CT and demonstrates the additional clinical importance of identifying multiple lesions. In patients with known or suspected CCM who present with neurological symptoms, brain MRI with susceptibility-sensitive sequences should be obtained regardless of the initial CT appearance. When MRI demonstrates multiple CCMs, familial disease should be considered, and genetic evaluation and appropriate family assessment should be pursued.

Management must be individualized. Conservative management is appropriate for selected stable patients, while microsurgical resection is particularly relevant to symptomatic, accessible CCMs and medically refractory CCM-related epilepsy. Stereotactic radiosurgery has a more restricted role, particularly for selected lesions in which microsurgical risk is excessive.

For the present patient, at the three-month follow-up, the absence of documented symptomatic hemorrhage, preserved neurological status, clinical stability under antiseizure treatment, and lack of a clearly concordant epileptogenic focus support continued conservative management. At the same time, genetic evaluation and detailed family assessment should form an important component of her long-term care, given the presence of multiple lesions and a potentially relevant family history.
